# Historic *Treponema pallidum* genomes from Colonial Mexico retrieved from archaeological remains

**DOI:** 10.1371/journal.pntd.0006447

**Published:** 2018-06-21

**Authors:** Verena J. Schuenemann, Aditya Kumar Lankapalli, Rodrigo Barquera, Elizabeth A. Nelson, Diana Iraíz Hernández, Víctor Acuña Alonzo, Kirsten I. Bos, Lourdes Márquez Morfín, Alexander Herbig, Johannes Krause

**Affiliations:** 1 Institute for Archaeological Sciences, University of Tübingen, Tübingen, Germany; 2 Senckenberg Center for Human Evolution and Paleoenvironment, University of Tübingen, Tübingen, Germany; 3 Institute of Evolutionary Medicine, University of Zurich, Zurich, Switzerland; 4 Department for Archaeogenetics, Max Planck Institute for the Science of Human History, Jena, Germany; 5 Molecular Genetics Laboratory, National School of Anthropology and History, Mexico City, Mexico; 6 Osteology Laboratory, National School of Anthropology and History, Mexico City, Mexico; McGovern Medical School at UTHealth, UNITED STATES

## Abstract

*Treponema pallidum* infections occur worldwide causing, among other diseases, syphilis and yaws. In particular sexually transmitted syphilis is regarded as a re-emerging infectious disease with millions of new infections annually. Here we present three historic *T*. *pallidum* genomes (two from *T*. *pallidum* ssp. *pallidum* and one from *T*. *pallidum* ssp. *pertenue*) that have been reconstructed from skeletons recovered from the Convent of Santa Isabel in Mexico City, operational between the 17^th^ and 19^th^ century. Our analyses indicate that different *T*. *pallidum* subspecies caused similar diagnostic presentations that are normally associated with syphilis in infants, and potential evidence of a congenital infection of *T*. *pallidum* ssp. *pertenue*, the causative agent of yaws. This first reconstruction of *T*. *pallidum* genomes from archaeological material opens the possibility of studying its evolutionary history at a resolution previously assumed to be out of reach.

## Author summary

Among the worldwide prevalent treponemal diseases syphilis is a global threat that is currently re-emerging. The origins of syphilis and other treponemal diseases are as yet unresolved and are subject to an intensive scholarly debate. Until now, assumptions on its origins and evolutionary history could only be drawn from osteological analyses of past cases and genetic analysis of contemporary *T*. *pallidum* genomes; contributions from ancient DNA are very rare and have so far failed to provide genome-level data. The ancient *T*. *pallidum* genomes presented here allow us, for the first time, to perform genome-wide comparative analyses and to assess a connection between osteological manifestations of past treponemal cases and specific *T*. *pallidum* species. Our study demonstrates the possibility of retrieving ancient *T*. *pallidum* genomes from archeological material and thereby establishes a new method that could greatly contribute to uncover the mystery regarding the origins of treponemal diseases.

## Introduction

*Treponema pallidum* subspecies cause several diseases, among which sexually transmitted syphilis, caused by *T*. *pallidum* ssp. *pallidum* (TPA), and yaws, caused by *T*. *pallidum* ssp. *pertenue* (TPE), are the best known and are prevalent worldwide. The global disease burden is high for both diseases. Syphilis is seen as re-emerging in various regions of the world including Europe, North America, China and Australia [1] with 10.6 million cases reported in 2009 [2], while for yaws more than 300,000 new cases were recorded between 2008 and 2012 [3]. The origins and evolutionary history of these pathogens remain nebulous, including the perceived sudden appearance and pandemic spread of syphilis in Europe at the end of the 15^th^ century. Hypotheses surrounding the origin of syphilis are subject to an extensive scholarly debate, in particular the New World origin of syphilis with worldwide dissemination starting in the 15^th^ century [4, 5] in contrast to the hypothesis of multiregional origin that posits an increase in virulence followed by a pandemic spread in the 15^th^ century [6]. While genetic data from contemporary *T*. *pallidum* strains has been interpreted as support for the New World origin [7, 8], there exists potential skeletal evidence in support of the multiregional origin [9, 10], yet this remains heavily debated [11, 12].

Ancient DNA could help to resolve this controversy by illuminating the evolutionary history of human-pathogenic treponemes. However, detection of ancient treponemal DNA is very rare. To date, only short strain-unspecific PCR fragments have been retrieved from a 200-year-old mummy [13] and from European post-Columbian neonates [14]. Furthermore, even in modern patients with advanced stage syphilis, who may display bone lesions, molecular detection of the bacterium is challenging [15] casting doubt on the possibility of a successful recovery of *T*. *pallidum* from ancient skeletons [16]. Our latest research on non-human treponemes, however, was successful in retrieving *T*. *pallidum* ssp. *pertenue* DNA from non-human primate bones that were several decades old [17].

Here, we use DNA hybridization capture methods in combination with high-throughput sequencing to retrieve historic *T*. *pallidum* DNA (*T*. *pallidum* ssp. *pallidum* and *T*. *pallidum* ssp. *pertenue*) and to successfully reconstruct three genomes using bone material dating back to Colonial Mexico with characteristic skeletal manifestations for congenital treponematosis [18]. Our study establishes the possibility of retrieving ancient *T*. *pallidum* genomes from archeological material and allows us, for the first time, to assess the genomic principle of past treponemal infections.

## Results

### Archeological site, dating and osteological analysis of the bone material

**Fig 1:**
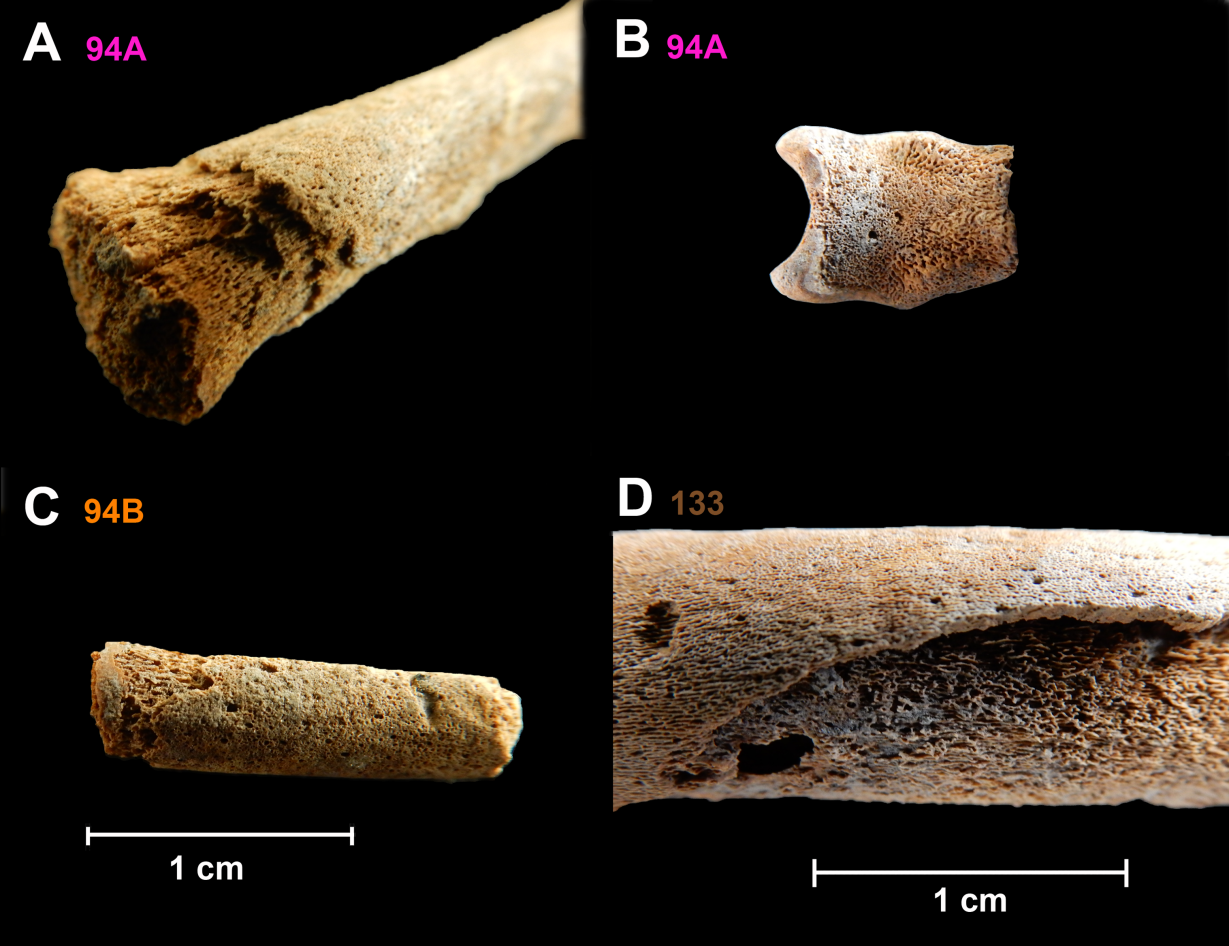
Examples for bone lesions for the three positive individuals. (A) The right tibia of individual 94A displays reactive periosteal bone on the anterior aspect of the diaphysis accompanied by progressive layering of the reactive bone. (B) The *pars basilaris* portion of the cranium of individual 94A showing pathological reactive bone in the endocranial surface, active at time of death. (C) An unidentified long bone from individual 94B displays fulminating periosteal reaction involving the whole of the diaphysis fragment. (D) The left femur of individual 133 presenting periosteal bone formation and expansion with cortical resorption characteristic of treponemal diseases. Source of the pictures: skeletal collection from Santa Isabel Convent, Mexico City, in custody of the Laboratory of Osteology, Post Graduate Studies Division, National School of Anthropology and History (ENAH), Mexico.

We screened samples from five individuals from the former Convent of Santa Isabel, a historical site located in downtown Mexico City used by nuns of the Franciscan Order from 1681 to 1861 [19]. Burials of 239 individuals (90% of which were foetuses, neonates and infants) were excavated from the remains of the convent in the 1990s, which were buried in the niches and tombs [19]. Five individuals were selected for this study based on the appearance of skeletal changes consistent with treponemal infection at the time of death. Of the five individuals, only three individuals were positive for treponemal DNA: individual 94A, 94B and 133 (Fig 1, S1 Fig). We estimated the age of these three individuals using a method based on dentition, as dental development is considered a more reliable indicator of age than developmental or degenerative skeletal changes [20]. The dental aging chart established by Ubelaker [21] was developed using American indigenous populations; however, when evaluating dental aging methods with a population of known ages, the Schour and Massler atlas [22] was found to have a higher accuracy of age estimations in individuals in below the age of 5.4 years [23]. Both methods were, therefore, employed to estimate the age of individuals as recommended previously [20, 21]. Our results reveal one perinatal individual (94B) and two infants (94A and 133) [21, 24]. Archaeological context as well as pH—EH quantification studies suggest a burial date during the Colonial period [19]. Radiocarbon dates of the individuals 94B and 133 suggest that both individuals were buried less than 350 years ago (younger than cal AD 1654 for 94B and younger than cal AD 1669 for 133).

These individuals present signs of infection in the form of abnormal skeletal changes such as periosteal reactive bone formation and diaphyseal expansion of long bones (Fig 1). Pathological skeletal changes were observed macroscopically and descriptively recorded as outlined by Buikstra and Ubelaker [25]. We recorded abnormal bone formation and bone destruction, status at time of death (active or healing) and location and extent of the affected bone. The resulting data was referenced to paleopathological and clinical literature [25–31]. The individuals displayed varied skeletal completeness and differential preservation due to taphonomic damage. The most consistent skeletal feature suggestive of infection present in the three individuals was periostitis, which included a multilayered periosteal reaction and dactylitis (Fig 1)[32–35]. Periostitis, though not specific to treponemal disease is strongly associated with treponematosis. It has been described as a “third category” of skeletal indicators of treponematosis and is present in greater frequency compared to other changes associated with treponemal disease such as cranial and dental changes [27]. Although these individuals (S1 Fig) do not display pathognomonic dental changes such as Hutchinson’s incisors or “mulberry” (or Founier’s) molars which are known to have a broad occurrence (33% and 27% respectively) in individuals bearing a congenital infection of syphilis, they do display abnormal periosteal bone development of the long bones and cranial bones [28]. To our knowledge, dental features associated with congenital syphilis are restricted to permanent teeth and would only be confidently visible around 6–9 months of age as only the sites of initiated ameloblast activity are visible before then [27, 36]. Although each individual was represented by a differing number of skeletal elements, all individuals presented layered periostitis. Individual 94A, estimated to be a six month-old infant based on skeletal development [24], displays a systemic, fulminating periosteal reaction present on the axial as well as the appendicular skeleton as illustrated by the severely affected tibia in Fig 1. Also present in this individual was dactylitis (expansive periostitis of the metacarpals), which is associated with treponematosis in the first year of life [27], among other conditions [26]. Furthermore, this individual displays abnormal reactive bone on the endocranial surface of the pars basilaris (Fig 1). Individual 94B is represented by fewer skeletal elements; however, periostitis suggestive of treponemal infection is still present on the ribs and an unidentified long bone (Fig 1). Based on the crown formation of the incisors and molars this individual is estimated to have died at birth +/- 2 months. Individual 133 presents bilateral periosteal reaction appearing to be active at time of death with extensive involvement of the appendicular and axial skeleton. As illustrated in Fig 1, the right femur of this individual displays significant periosteal bone formation with some cortical resorption. This individual is represented by a nearly complete, albeit fragmentary, skeleton, with four mandibular molars, two maxillary incisors, and two canines. Dental elements from the individual were used to estimate the age at death as six months (+/- 3 months) post partum [21].

The skeletal changes displayed by the three individuals suggest treponemal infection that is possibly congenital given their young ages [26, 37]; however, the nature of the skeletal changes, being non-specific yet suggestive of treponemal infection emphasizes the importance of analysis through molecular paleopathological methods. Ancient DNA provides the possibility to both identify a causative infectious organism and explore its ecological and evolutionary history.

### Genome-wide enrichment, assembly and analysis of the *T*. *pallidum* DNA

Long bones with lesions suggestive of treponemal infections were sampled for all five individuals; ancient DNA was extracted [38] and double stranded next generation sequencing libraries were constructed for all samples [39, 40]. To reduce the background of environmental DNA, whole-genome array capture for *T*. *pallidum* subspecies [41] was conducted. After Illumina sequencing to a depth of 98 to 204.5 million pre-processed pair-end reads, mapping to the reference genome (NC_021490.2) and duplicate removal, between 71,524 and 128,419 unique DNA sequencing reads were retrieved for individuals 133, 94A and 94B, resulting in an average of 3.25 to 7.72-fold genome coverage with a duplication factor of 28.64 to 37.25 and 57–94% of the reference genome covered at least 3-fold (Table 1).

**Table 1:** Summary statistics of the three positive historic samples.

Individual	94A		94B		133	
DNA analyzed	treponemal	mitochondrial	treponemal	mitochondrial	treponemal	mitochondrial
**Mapped reads**	74827	21074	128419	29157	71524	32142
**Average coverage**	4.4125	93.44	7.7267	154.50	3.2514	160.00
**Genome coverage ≥ 3fold**	74.31%	99.98%	93.42%	100%	57.28%	100%
**DNA damage 1^st^ base 5’**	0.1098	0.1159	0.1029	0.1098	0.1674	0.1582
**DNA damage 1^st^ base 3’**	0.1125	0.1196	0.0993	0.0956	0.1753	0.168
**Contamination**	NA	0.002	NA	0.002	NA	0.002
**Haplogroup**	NA	D1i2	NA	D1i2	NA	H1c+152

Mapping statistics reported by the EAGER pipeline for the three historic samples. The table includes total number of mapped reads, average coverage, percent of the genome covered at 3 fold coverage and the DNA damage for the 1^st^ base at the 5’ end and 3’ end for both treponemal and human mitochondrial DNA. In addition, the estimated amount of contamination and the haplogroup is added for the human mitochondrial DNA.

### Analysis and authenticity of the retrieved *T*. *pallidum* and human DNA

To assess the authenticity of the captured *T*. *pallidum* DNA we analyzed nucleotide misincorporation patterns characteristic of ancient DNA [42, 43]. The observed DNA damage patterns of 10% to 17% C to T damage at the terminal base of the DNA fragments support the ancient origin of the retrieved *T*. *pallidum* DNA (Table 1). Human mitochondrial fragments were simultaneously enriched via a streptavidin-based bead capture [44] resulting in complete mitochondrial genomes with haplogroups found today in Central America (D1i2 for 94A & 94B and H1c+152 for 133). Consistent with the ancient pathogen, 11% to 17% DNA damage was observed at the terminal ends, thus supporting their authenticity (Table 1). Contamination of the human mitochondrial DNA calculated using the program Schmutzi [45] in all three samples was estimated at less than 2% (Table 1).

### Phylogenetic analysis of the historic *T*. *pallidum* genomes

**Fig 2:**
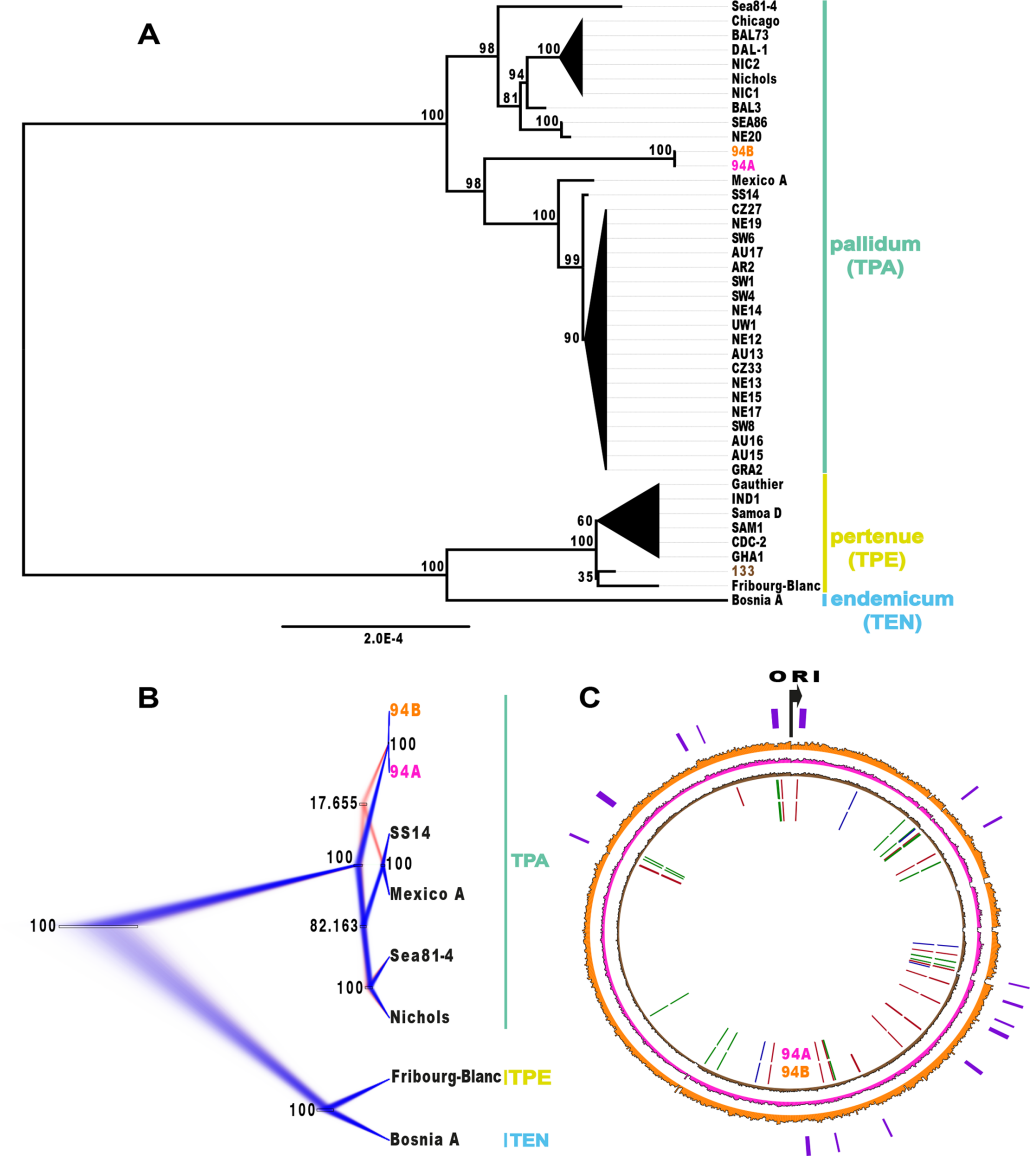
Phylogenetic trees and Circos plot [90] of the three ancient strains in comparison to modern strains. (A) Maximum Likelihood tree with bootstrap support for 39 modern strains and the three ancient strains. The strains 94A (magenta) and 94B (orange) branch with the syphilis SS14 clade while strain 133 (brown) branches with Fribourg-Blanc and other yaws strains. The scale represents the mean number of substitutions per site according to the GTR+GAMMA. Colored bars highlight the three subspecies *Treponema pallidum* ssp *pallidum* (TPA), *pertenue* (TPE) and *endemicum* (TEN). Strains of subspecies *pallidum* cause syphilis, subspecies *pertenue* cause yaws and subspecies *endemicum* causes bejel. (B) Bayesian trees visualized in Densitree overlaying phylogenetic trees based on the most probable topologies. Blue colored trees represent the most probable topology followed by red colored trees. For the ancient strains 94A and 94B two conflicting topologies are visible. The bars represent the 95% highest probability density intervals of the heights of the clades. The support value given at each clade is the fraction of trees in the tree-set that contain the clade. (C) Circos plot showing the shared SNP positions with specific clades and the coverage of the three ancient strains. From outer circle to the inner circle regions of possible recombination detected by ClonalFrameML are denoted on the outermost circle (purple). ‘ORI’ refers to the origin of replication. The genome coverages of the ancient strains 94B, 94A and 133 are represented in orange magenta and brown respectively from outward to inwards. Based on the SNPs that are specifically shared with different clades, colored bars are shown for strains 94B and 94A respectively in the innermost circles. Red bars highlight the SNP positions specifically shared with Fribourg-Blanc (supporting a phylogenetic position ancestral to the two syphilis clades). The green bars highlight the SNP positions shared with the SS14 clade while the blue bars highlight the SNP positions shared with the Nichols clade.

We reconstructed a phylogeny for the three historic and 39 publicly available modern *T*. *pallidum* genomes [41] using different reconstruction methods. Our Maximum Likelihood (Fig 2A) and Maximum Parsimony (S2A Fig) trees position two of the historic strains (94A, 94B) on the branch of the modern syphilis-causing *T*. *pallidum* ssp. *pallidum* strains, while the third historic strain (133) falls with the modern yaws-causing *T*. *pallidum* ssp. *pertenue* strains. A precise placement of sample 133, however, within the yaws clade cannot be further resolved owing to its lower coverage. The strains 94A and 94B are very similar and differ only in one single position unique to 94A (237,661) in the gene TPANIC_RS01145 coding for a cobalt ABC transporter ATP-binding protein. Twenty-seven SNP positions are unique to 94A and 94B while three SNP positions are specific to sample 133 (S2 Table). In the Maximum Likelihood tree they branch within the SS14 clade, while they fall basal on the syphilis branch in the Maximum Parsimony tree with reduced bootstrap support. This phenomenon could be linked to DNA mosaic patterns, such as those identified previously in three *T*. *pallidum* strains [46–48], leading to the suggestion that *T*. *pallidum* strains may horizontally recombine [49].

### Analysis of ambiguous SNP positions in the historic genomes

To further investigate this phenomenon we performed several analyses. We constructed a Bayesian phylogenetic tree from 1,061 SNP positions after deletion of positions with missing data from a SNP alignment of the historic strains 94A and 94B with Fribourg-Blanc, Bosnia_A, Nichols, Sea81-4, Mexico_A and SS14 using BEAST2 [50]. The uncertainty in the branching pattern of 94A and 94B was visualized using Densitree [51] by overlaying the different tree topologies from the BEAST2 run (Fig 2B). We found the highest support for an ancestral placement in 82.16% of the tree states (Fig 2B, blue color). Only 17.66% of the tree states (Fig 2B, red color) support the branching of the historic strains within the SS14 clade, and 0.18% of the tree states support a trifurcation of the 94A and 94B strains, the SS14 clade (SS14, Mexico_A) and the Nichols clade (Nichols, Sea81-4).

To study the derived SNPs that the historic strains share with different clades in more detail we also conducted a comparative SNP analysis using 2,294 SNPs among a complete set of 42 *T*. *pallidum* strains and identified the following: for 94A and 94B, 22 SNPs and 30 SNPs, respectively, support an ancestral position (Fribourg-Blanc clade), 19 and 24 SNPs, respectively, support a position in the SS14 clade, and 4 and 5 SNPs, respectively, support a position in the Nichols clade (Fig 2C). In general, the loci of these shared SNPs are spread across the genome and are not specific to certain regions.

ClonalFrameML [52] showed evidence of recombination as previously described [41]: depending on the applied model we detected between 13 (standard model) and 16 (per-branch model) predicted recombination events corresponding to the common ancestor of 94A and 94B (S3 Table). The combination of recombination events from both models (standard and per-branch) was analyzed by comparing these regions to SNP positions that 94A and 94B share with different clades (S1 Table). Most of the regions are shared with Fribourg-Blanc and thereby further support the ancestral positioning of the historic strains. We also found one recombination event (346215–347572; TPANIC_RS01590) that is congruent with previous reports on recombination in two genes (TPANIC_RS01590, TPANIC_RS02370) in the contemporary Mexico_A strain [46]. Interestingly, for TPANIC_RS02370 no recombination event was observed in the common ancestor of 94A and 94B pointing to the possibility of a recombination event that happened later in time. We identify multiple regions where the common ancestor of 94A and 94B shows putative recombination events with the ancestors of TPA (*pallidum* subspecies), TPE (*pertenue* subspecies) and TEN (*endemicum* subspecies) suggestive of inter-strain recombination as has been proposed earlier [46–48]. Furthermore, the genes TPANIC_RS04240, TPANIC_RS04770 and TPANIC_RS05095, which were previously proposed to be recombining [47–49], were also detected as part of potentially recombining regions in our analysis (S3 Table).

To assess the effects of these findings in the context of single-gene phylogenies, we tested different model topologies (S2B and S2C Fig) on 1,028 genes in a TREE-PUZZLE analysis [53]. Out of 1,028 genes, 544, 564 and 509 genes in strains 94A, 94B and 133 have at least one informative SNP in the gene alignment, of which 221, 180 and 246, respectively, do not reject any of the model trees. Among the remaining genes, 199 genes of strain 133 rejected any positioning of the strain within the syphilis clades, while for strains 94A and 94B, 265 and 322 genes, respectively, reject any position within the yaws clade. For all remaining genes, the rejected tree topologies are listed in S1 Table. We found the highest support for an ancestral placement of 94A and 94B with only seven and eight genes rejecting this positioning (S1 Table). For the third historic strain, 133, its positioning in the yaws clade has the highest support only rejected by one gene (*proA*; TPANIC_RS01715), which contains a SNP (position 376,586) that is specifically shared with Nichols (S1 Table).

### Virulence factor analysis

The presence of 61 genes previously thought to be related to virulence based on other studies was evaluated in the ancient and modern strains. This included the Tpr family, various outer membrane proteins, adhesion proteins, lipoproteins and a few other classes [54–60]. The ancient strains identified as syphilis harbor all of these factors except TPANIC_RS04235 a homolog of the FadL family that enables the transport of long-chain fatty acids (LCFAs) as well as other hydrophobic nutrients [61]. In general, *Treponema pallidum* seems to harbor altogether five FadL orthologs [62], of which the other four are present in the ancient strains. In addition, previous studies have described a deletion of the gene TPANIC_1030 in the *pertenue* strains [41, 63], which is also confirmed in the ancient *pertenue* strain 133 (S3 Fig and S4 Table).

## Discussion

The origin of syphilis and the evolutionary history of treponemal diseases are subjects of on-going debate. Ancient *T*. *pallidum* genomes have the potential to provide evidence to address many questions raised in this context. Here we provide the first historic genomes of *T*. *pallidum* subspecies. Our data is of sufficient resolution to allow for a global genomic and phylogenetic analysis. Although our historic genomes cannot directly contribute to discussions about the origin of syphilis due to their post–Columbian age, our study demonstrates the potential of successfully obtaining authentic historic *T*. *pallidum* genomes when focusing on treponemal cases in young individuals. More historic *T*. *pallidum* genomes, in particular from the pre-Columbian era, could be able to settle the debate. However, the selection of samples, retrieval of ancient *T*. *pallidum* DNA and comparative genomic analyses pose multiple challenges.

We find strong evidence that the bone lesions in two historical treponematosis cases, one in a perinate and another in an infant, were caused by *T*. *pallidum* ssp. *pallidum*. Furthermore, we present skeletal and genomic evidence of *T*. *pallidum* ssp. *pertenue* infection in an infant individual estimated to be six months (+/- 3 months) of age at time of death. This individual calls to attention the possible existence of congenital yaws, which has been debated in the literature due to the apparent inability of *T*. *pallidum* ssp. *pertenue* to cross the placental barrier, despite the description of potential historical examples [64, 65]. Variation in the ability for vertical transmission has been demonstrated within *T*. *pallidum* subspecies, for example the Haiti B strain of *T*. *pallidum* ssp. pallidum also seems unable to cross the placenta [66, 67] suggesting the possibility of such variation within the *pertenue* subspecies strains. Thus, the debate of whether *T*. *pallidum* ssp. *pertenue* can [64, 65], or cannot [67, 68] cross the placental barrier to cause a form of congenital treponematosis remains unresolved. The severity of the skeletal response in individual 133 together with the young age at death (6 months, +/- 3 months) supports a diagnosis of congenital yaws; however, the known pathophysiology of *T*. *pallidum* ssp. *pertenue* infections, namely that skeletal changes can occur in infants infected up to four months post partum [64, 65], challenges an unequivocal diagnosis of congenital yaws for this case and highlights the importance of further attention to this research question.

Although the individuals included in our study present skeletal changes consistent with but not limited to treponematosis, producing a differential diagnosis of a greater resolution was not possible due to the shared signs of yaws and syphilis and the differential preservation of the skeletons, which compromised the ability to visualize diagnostic patterning of the pathological changes. Likewise, such challenges exist in the diagnosis of clinical cases whereby evidence of the overlapping modes of transmission and clinical features of syphilis and yaws can be found in biomedical research on modern *T*. *pallidum* strains, such as *T*. *pallidum* ssp. *pallidum* causing yaws-like frambesiform lesions [69] or *T*. *pallidum* ssp. *pertenue* invading neurological and cardiovascular tissue as seen in syphilis [64, 70]. However, our genetic results allowed us to identify syphilis infection in two of the individuals (94A, 94B) whereas one individual (94B) presents a likely congenital infection of syphilis. In addition, we identified yaws in one infant individual (133). Our work demonstrates the value of molecular identification of ancient pathogens, particularly as applied to treponemal diseases where skeletal responses to the various pathogenic subspecies are often shared, challenging the development of a confident diagnosis through osteological observation [27]. Molecular analyses not only provide greater resolution to the paleopathological diagnosis of osteological changes but they also provide relevant details related to the history and evolution of pathogens we still encounter today.

In the phylogenetic comparison of our historic strains with contemporary *T*. *pallidum* strains, we detected ambiguous SNP patterns in our strains that may suggest a number of recombination events in its evolutionary past. Although *T*. *pallidum* subspecies are thought to be clonal [71], several studies on modern treponemal DNA have reported evidence for recombination events in various *T*. *pallidum* subspecies [41, 46–48]. As recombination mechanisms are active during treponemal infections, TPA strains could have integrated some genomic regions from TPE strains during a co-infection of the same host [46, 48]. The presence of yaws in sample 133 and syphilis in samples 94A and 94B indicates that *pertenue* and *pallidum* infections were both prevalent among individuals in this region, which could have opened the possibility of recombination events facilitated by co-infections with these subspecies. Further research on ancient and modern *T*. *pallidum* strains is needed to assess this aspect of their evolution, which should be borne in mind in phylogenetic assessments.

Various studies on the pathogenicity of treponemes have suggested a number of genes potentially involved in virulence, most of which encode proteins that reside at the host-pathogen interface. Regarding the presence and absence of these genes we did not detect a difference in our ancient strains compared to modern ones with the exception of the FadL homolog TPANIC_RS04235, which is absent in our ancient syphilis strains. FadL facilitates the transport of long-chain fatty acids (LCFA), which are metabolic energy sources and can also aid in pathogenesis. Uptake of high concentrations of LCFAs released by host cells would assist in suppression of inflammatory responses thereby enabling the pathogen to colonize the host more efficiently [72, 73]. However, there are four other FadL homologs present and it is unclear if they target different compounds with varying specificity [57]. Thus, it currently remains an open question if the absence of one of the FadL homologs could influence phenotype.

In conclusion, our study demonstrates that retrieval of authentic ancient *T*. *pallidum* DNA from historic tissues to the resolution of genomic reconstruction is indeed possible, despite earlier pessimism [74]. The presence of *T*. *pallidum* ssp. *pertenue* in old world monkeys [17, 75] and our finding that two *T*. *pallidum* subspecies likely caused similar osteological manifestations in the past may suggest a more complex evolutionary history of *T*. *pallidum* than previously assumed. Furthermore, our detection of possible recombination events in the past highlights an important, and currently underrepresented, analytical component that should be accommodated in future models of *T*. *pallidum*'s history. Our results point out the necessity of ancient *T*. *pallidum* genome-level data to better understand both osteological manifestations caused by infection of the various forms and their potential for genomic recombination in the past. Ultimately, these analyses may be able to resolve the origin of syphilis and the evolutionary history of treponemal diseases in general.

## Methods

### DNA extractions and library preparation

For all five samples, DNA from 50 mg of bone powder was extracted in clean room facilities dedicated to ancient DNA work at the University of Tübingen using a silica purification protocol [38] with the following modifications: the Zymo-Spin V funnels (Zymo Research) were bleached and UV irradiated for 60 minutes and the total elution volume was raised to 100 μl. To obtain double-stranded Illumina libraries, aliquots of 20 μl of extract were used in a well-established protocol [39] and sample-specific barcodes were added to both library adapters by amplification after adapter ligation [39, 40]. For all samples, two sets of double-stranded Illumina libraries were created: with and without UDG treatment [76]. Extraction and library blanks were treated accordingly. After quantification of the indexed libraries using the IS5 and IS6 primer set [39], the DyNAmo Flash SYBR Green qPCR Kit (Biozym) and the Lightcycler 96 (Roche), a second amplification was carried out in 100 μl reactions for each library with 5 μl of library template, 4 units of AccuPrime Taq DNA Polymerase High Fidelity (Invitrogen), 1 unit of 10X AccuPrime buffer (containing dNTPs) and 0.3 μM of IS5 and IS6 primers [39] and the following thermal profile: 2-min initial denaturation at 94°C, followed by 6 to 16 cycles consisting of 30-sec denaturation at 94°C, a 30-sec annealing at 60°C and a 2-min elongation at 68°C and a 5-min final elongation at 68°C. Subsequently, a purification using the MinElute PCR purification kit (Qiagen, Hilden, Germany) and quantification with Agilent 2100 Bioanalyzer DNA 1000 chips was performed.

For the three samples positive for *T*. *pallidum* DNA (94A, 94B and 133), a second extract from 50 mg of bone powder as described above and additional single-stranded libraries were created [77], and the previously described procedures were followed after the library preparation step.

### Genome wide enrichment and sequencing of the *T*. *pallidum* DNA

The genome wide enrichment for *T*. *pallidum* DNA was performed via two rounds of hybridization capture using a one-million-feature Agilent microarray and a well-established protocol [78]. The design of the microarray was previously detailed by Arora and coauthors [41]. After array enrichment the libraries were amplified, purified and quantified as described above.

Paired-end dual index sequencing was carried out on an Illumina HiSeq 2500 platform with 2*100+7+7 cycles using the manufacturer’s protocols for multiplex sequencing (TruSeq PE Cluster Kit v3-cBot-HS).

For the positive samples a second enrichment including an additional set of single-stranded libraries and further sequencing was performed as detailed before.

### Processing of the sequencing reads and reconstruction of the *T*. *pallidum* genomes

Paired-end reads from the three ancient samples, 28 modern samples [41] and simulated reads (reads of length 100 and offset 1) from 11 complete genomes were pre-processed, mapped and post-processed using the EAGER pipeline version 1.92 [79]. After trimming of adaptors and filtering for reads with a minimum base quality of 20 and a minimum read length of 30bp, overlapping forward and reverse reads were merged. These merged and unmerged forward and reverse reads were mapped to the *T*. *p*. ssp. *pallidum* Nichols (NC_021490.2) complete genome as a reference with BWA [80] using a mapping stringency of 0.1 (-n) and a mapping quality filter of 37. Duplicated reads were eliminated using PICARD tools Mark Duplicates [81]. Reads from non-UDG treated libraries were used to estimate the authenticity of the ancient DNA samples. DNA damage in the ancient samples was quantified using MapDamage 2.0 [82]. SNPs were called using UnifiedGenotyper of the Genome Analysis Toolkit (GATK) [83]. These resulting VCF files were combined and compared using an in-house tool (MultiVCFAnalyser). Reference bases and SNPs were called with a minimal coverage of 3 and a minimal major allele frequency of 90%. Calls below this threshold are represented as “N”. The annotation and effect of SNPs within protein-coding regions are identified using SnpEff [84]. All SNPs were concatenated for phylogenetic analysis and downstream analysis.

### Enrichment and sequencing of the human mtDNA

Along with bacterial DNA, human mitochondrial DNA from the three ancient samples was sequenced. The paired-end reads were mapped to the HG19 mitochondrial genome (NC_012920.1) using the EAGER pipeline version 1.92 [79]. MapDamage 2.0 [82] was used to estimate DNA damage. Estimation of contamination with modern DNA and generation of consensus sequences was performed using Schmutzi version 1.0 [45]. Haplogroups were determined using Haplogrep 2 [85].

### Phylogenetic analysis of the *T*. *pallidum* genomes

The concatenated full genome alignment (equal in length to the reference NC_021490.2) of all 42 samples (includes the three ancient samples) was used to assess the relationship of the ancient samples with modern treponemes. Phylogenetic trees were built using RAxML [86] and MEGA 7.014 [87]. Maximum Likelihood trees were built with the GTR+GAMMA+I substitution model with a proportion of Invariant sites of 0.01 with 1000 bootstrap replicates. Evolutionary rates of the sites were estimated based on Gamma distributions with 8 categories. Maximum Parsimony trees were generated for all sites of the full genome alignment using the Subtree-Pruning-Regrafting (SPR) search method and 1000 bootstrap replicates. BEAST2.4.2 [50] was used to reconstruct phylogenies for the SNP alignment of 94A, 94B and selected representative genomes (Nichols, Sea81-4, SS14, Mexico_A, Fribourg-Blanc, BosniaA) after complete deletion of sites with missing data. Bayesian phylogenetic tree states were generated with a GTR-based substitution model with 8 Gamma categories and the proportion of invariant sites estimated from the data. Substitution frequencies of all nucleotide pairs are estimated with CT pair initial frequency of 1.0. A strict clock model was set with an initial rate of 0.005. The priors for the trees were set to Coalescent Constant Population model. All default parameters were used for priors after setting the population model. For the MCMC runs the chain length was set for 1,000,000,000 steps with a burn-in of 10% (100,000,000) and cataloguing tree states and log information for every 10,000 steps. Results were analyzed for stability using Tracer version 1.6 [88]. All the trees were visualized using Densitree version 2.2.5 [51].

### Detection of mosaic patterns in the *T*. *pallidum* genomes

The contribution of SNPs of the ancient samples was analyzed in detail to resolve the uncertainty of topological differences in the phylogenetic trees using Maximum Likelihood and Bayesian approaches. SNPs from ancient samples 94A and 94B were compared with Fribourg-Blanc (representing the Yaws clade), Nichols (representing the Nichols clade) and SS14 (representing the SS14 clade) using an in-house tool (MultiVCFAnalyser). SNPs supporting the ancestral position are SNPs that are shared specifically with Fribourg-Blanc (ancestral SNPs) but are derived in SS14 and Nichols. SNPs supporting the SS14 position are SNPs that are shared specifically with SS14 (derived SNPs) but are ancestral in Fribourg-Blanc and Nichols. SNPs supporting the Nichols position are SNPs that are shared specifically with Nichols (derived SNPs) but are ancestral in Fribourg-Blanc and SS14.

ClonalFrameML version 1.-178 [52] was used to predict recombination patterns in the ancient strains. Using a Maximum Likelihood tree generated with RAxML as the initial tree and the full genome alignment of 42 samples (three ancient samples included), recombination events were predicted by using the standard model of the Baum-Welch expectation maximization algorithm (EM) for 1000 iterations with default parameters. These parameters were applied to all branches. The recombination parameters were also estimated for each individual branch (per-branch model) with a variability of recombination parameters of 0.1. The predicted recombination events from the standard run and the per-branch run were compared and combined.

For more in depth analysis of any ambiguity in branching patterns that contradict the Maximum Likelihood phylogenetic tree we used TREE-PUZZLE version 5.3 [53]. It compares various models of tree topologies to the constructed maximum likelihood tree and finds the rejected tree models using various statistical tests. For each of the three ancient samples an individual full genome alignment fasta file was generated (equal in length to the reference NC_021490.2) with Nichols, Sea81-4, SS14, Mexico_A, Bosnia_A, and Fribourg-Blanc using an in-house tool (MutliVCFAnalyser). All ambiguous bases are denoted as “N” in the full genome alignment. For each gene, the alignment spanning the gene coordinates from the NC_021490.2 annotation file was extracted and modified to phylip 3.0 format. TREE-PUZZLE [53] was then used to reconstruct phylogenetic trees from the gene alignments and to compare them with our nine pre-defined model tree topologies. With the Fribourg-Blanc, Bosnia_A, Nichols, Sea81-4, SS14 and Mexico_A taxa, a backbone tree topology was created using RAxML. Independently, 94A, 94B and 133 were placed at every possible position on the tree backbone to generate nine model trees that were used to reconstruct the Maximum Likelihood tree from the gene alignment (S2B Fig). Model trees 1, 2, 6, 7, 8 and 9 represent the branching of 94A, 94B and 133 with Fribourg-Blanc, Bosnia_A, SS14, Mexico_A, Nichols and Sea81-4 respectively. Model tree 3 represents an ancestral branching point that splits the sexually transmitted treponemes (*T*. *p*. ssp. *pallidum*) and non-sexually transmitted treponemes (*T*. *p*. ssp. *pertenue* and *T*. *p*. ssp. *endemicum*). Model tree 4 represents a branching that is at a basal position within the SS14 clade (ancestral to the split of SS14 and Mexico_A). Model tree 5 represents a branching that is at a basal position within the Nichols clade (ancestral to the split of Nichols and Sea81). TREE-PUZZLE [53] generated a Maximum Likelihood tree with the GTR substitution model and evolutionary rate estimation using Gamma distribution with eight categories. The search procedure was set to evaluate the user-defined trees (nine model trees here). Initial Parameters were estimated using a Quartet sampling and neighbor-joining tree internally. TREE-PUZZLE generated a comparison table of statistical tests by comparing the topology of the nine gene trees reconstructed from the nine model trees. TREE-PUZZLE reports parsimoniously informative SNPs. For each gene, SNPs were identified and compared with the parsimoniously informative SNPs from TREE-PUZZLE. Genes without any SNPs were discarded and remaining genes were evaluated. Using Expected Likelihood Weight (ELW) statistics, a phylogenetic gene tree was considered rejecting any of the nine model trees based on a threshold of 0.05. TREE-PUZZLE was run for 1028 genes. For robust results, the process was repeated 100 times with the same parameters for 94A, 94B, 133.

### Virulence factor analysis

A gene presence/absence analysis was performed to assess the virulence factors harbored by the ancient genomes. A set of 61 gene sequences potentially related to virulence [55–57] were extracted from the Nichols reference genome (NC_021490.2) with 200 bases upstream and downstream (S4 Table). Reads from the 42 genomes including the ancient genomes were mapped to the gene sequences of the set with a mapping quality threshold of 0 using BWA. Read duplicates were removed using MarkDuplicates as described above. The coverage over each gene was calculated using genomeCoverageBed in BEDTools version 2.250 [89]. Gene coverage by the reads is estimated at 1x and visualized using ggplots2 package in R (S3 Fig).

### Ethic statement

Research on historic human remains presents the ethical problem that informed consent can no longer be obtained from the individuals on whom the research is being conducted. In many cases it is also impossible to identify down close relatives that would be able to provide an informed consent to conduct genetic or anthropological research of their ancestors. Our research, however, does not include culturally sensitive material such as Native American- or Australian Aboriginal populations, whereby it is necessary to acquire the informed consent of the descendants in order to conduct research. Furthermore, we focus on infectious diseases that potentially affect all human populations regardless of cultural identity. The samples analyzed in this manuscript are part of Santa Isabel collection in Mexico City. For this study we obtained a permit from the Council of Archaeology, National Institute of Anthropology and History (INAH, Mexico City, Mexico) to analyze the individuals from the Santa Isabel collection (official notice number: 401.B(4)19.2015/36/1027; approved partial report number: 401.1S.3-2017/1065). In addition, this work is also part of an ongoing research project on venereal and congenital syphilis identifying osseous remodeling and differential diagnostic led by Lourdes Márquez Morfín, approved by both the Council of Archaeology, National Institute of Anthropology and History (INAH, Mexico City, Mexico), and the Postgraduate Studies Division, National School of Anthropology and History (ENAH, Mexico City, Mexico).

## Supporting information

S1 Fig Full skeletons and features of interest for individuals 94A, 94B and 133 from Santa Isabel Convent. (A) Complete skeletal elements for individual 94A. (B) Metacarpals and phalanges of individual 94A displaying periostitis. (C) Complete skeletal elements for individual 94B. (D) Fragment of the still developing molar crown (at time of death) of individual 94B. (E) Complete skeletal elements for individual 133. (F) First mandibular molar of individual 133 displaying normal crown development. Source of the pictures: skeletal collection from Santa Isabel Convent, Mexico City, in custody of the Laboratory of Osteology, Post Graduate Studies Division, National School of Anthropology and History (ENAH), Mexico.
(JPEG)

S2 Fig Maximum Parsimony tree and TREE-PUZZLE analysis. (A) Maximum Parsimony tree with bootstrap support for 39 modern genomes and the three ancient samples. The scale bar represents the number of mutations over the whole genome calculated by average pathway method. Colored bars highlight the three subspecies *Treponema pallidum* subspecies *pallidum* (TPA), *pertenue* (TPE) and *endemicum* (TEN). Strains of subspecies *pallidum* cause syphilis, subspecies *pertenue* causes yaws and subspecies *endemicum* causes bejel. (B) The nine model trees used in the TREE-PUZZLE analysis. The colored edges represent the branching of the ancient sample with specific representative strains that are denoted by their names on the tree topology backbone. The red edge represents an ancestral branching of the historic sample while the green edge denotes the branching of the sample with the SS14 clade and the blue edge denotes the branching of the sample with the Nichols clade. The number in parenthesis represents tree topology model. (C) Bar plot representing the number of genes of the historic strains 94A, 94B and 133 that reject specific tree topologies. Each plot represents a specific tree model for 94A (magenta), 94B (orange) and 133 (brown) and the bar colors correspond to the color scheme as in S2B Fig.
(PNG)

S3 Fig Virulence factor analysis of ancient and modern genomes. The heatmap summarizes the presence and absence of various virulence factors for the ancient and modern genomes in terms of gene coverage. The color key represents the percentage of coverage. Red represents gene presence while green represents gene absence. The ancient strains 94A (magenta), 94B (orange) and 133 (brown) are highlighted. Colored bars represent the three subspecies subsp. *pallidum* (TPA), subsp. *pertenue* (TPE) and subsp. *endemicum* (TEN).
(PNG)

S1 Table Comprehensive listing of genes that contradict specific tree topologies. For strains 94A and 94B all genes are listed that reject tree topologies other than those that place the strains within the yaws clade. For strain 133 all genes are listed that reject tree topologies other than those that exclude all possible positions within the syphilis clade. In case a gene rejects varying sets of tree topologies during multiple TREE-PUZZLE runs the different patterns are listed (separated by '//'). If a gene does not reject any of the tree topologies for one of the strains the rejection pattern is denoted as '0'. SNP positions of 94A and 94B that are specifically shared with Fribourg (a representative of the Yaws clade), Nichols (a representative of the Nichols clade) or SS14 (a representative of the SS14 clade) are listed for affected genes. For each position, the alternative allele and the representative strain that shares this SNP position are represented for 94A and 94B. If multiple SNP positions from a gene contribute to the ambiguity, all the SNP positions are specified (separated by ';'). In cases were one of the two strains has missing data this is indicated by a '*'. The effect of the SNPs along with the nucleotide change (specified in sense strand direction) and codon change is specified as well as the annotated gene function. If a gene is within genomic regions predicted to be recombining by ClonalFrameML in strains 94A and 94B, the region is specified. Pseudogenes are labeled with '(a)'.
(XLSX)

S2 Table List of SNPs that are specific to 94A, 94B and 133. 27 unique SNPs were identified for 94A and 94B while three unique SNPs were identified in 133. The reference allele (Nichols) and the alternative allele, the gene, gene annotation and the effect of the variant are provided for each SNP. The position within the gene and its corresponding amino acid change is also provided.
(XLSX)

S3 Table List of recombination events as detected by ClonalFrameML. A list of 16 recombination events detected by ClonalFrameML in the common ancestor of the ancient genomes 94A and 94B is provided. For each region the length and number of SNPs within that region are provided. For each region the number of SNPs per base pair is given as a factor of the genome-wide average. For each region, recombination events occurring in both internal and terminal branches of the tree that are sharing the event with the common ancestor of 94A and 94B are specified. Genes located within the predicted recombinant regions are listed together with their annotated functions.
(XLSX)

S4 Table Virulence factor analysis. Overview of 61 potentially virulence related genes. The start and end coordinates and strand information of the genes are provided as well as RefSeq and GenBank locus tags.
(XLSX)

## References

[1] Stamm LV. Syphilis: Re-emergence of an old foe. Microbial cell (Graz, Austria). 2016;3(9):363–70. Epub 2017/03/31. doi: 10.15698/mic2016.09.523 ; PubMed Central PMCID: PMCPmc5354565. 28357375 10.15698/mic2016.09.523 PMC5354565

[2] World Health Organization. Global incidence and prevalence of selected curable sexually transmitted infections-2008: World Health Organization; 2012.

[3] Kazadi WM, Asiedu KB, Agana N, Mitjà O, Guinea PN. Epidemiology of yaws: an update. Clinical Epidemiology. 2014;6:119–28. doi: 10.2147/CLEP.S44553 24729728 10.2147/CLEP.S44553 PMC3979691

[4] Crosby A. The early history of syphilis: A reappraisal In The Columbian exchange: biological and cultural consequences of 1492, ch. 4. Westport (Connecticut): Greenwood Press; 1972 p. 122–64.

[5] Quétel C, Braddock J, Pike B. History of syphilis: Polity Press Cambridge; 1990.

[6] Hackett CJ. On the origin of the human treponematoses (pinta, yaws, endemic syphilis and venereal syphilis). Bulletin of the World Health Organization. 1963;29:7–41. 14043755 PMC2554777

[7] Harper KN, Ocampo PS, Steiner BM, George RW, Silverman MS, Bolotin S, et al On the origin of the treponematoses: a phylogenetic approach. PLoS neglected tropical diseases. 2008;2(1):e148–e. doi: 10.1371/journal.pntd.0000148 18235852 10.1371/journal.pntd.0000148 PMC2217670

[8] Gray R, Mulligan C, Molini B, Sun E, Giacani L, Godornes C, et al Molecular evolution of the tprC, D, I, K, G, and J genes in the pathogenic genus Treponema. Molecular biology and evolution. 2006;23(11):2220–33. doi: 10.1093/molbev/msl092 16926243 10.1093/molbev/msl092

[9] Blondiaux J, Bagousse A. A treponematosis dated from the Late Roman Empire in Normandie, France. L’Origine de la syphilis en Europe: Avant ou aprés. 1994;1493:99–100.

[10] von Hunnius TE, Roberts CA, Boylston A, Saunders SR. Histological identification of syphilis in pre-Columbian England. American Journal of Physical Anthropology. 2006;129(4):559–66. doi: 10.1002/ajpa.20335 16345063 10.1002/ajpa.20335

[11] Baker B, Armelagos G. The origin and antiquity of syphilis: paleopathological diagnosis and interpretation. Current anthropology. 1988;29(5):703 11613900 10.1086/203691

[12] Harper KN, Zuckerman MK, Harper ML, Kingston JD, Armelagos GJ. The origin and antiquity of syphilis revisited: an appraisal of Old World pre-Columbian evidence for treponemal infection. Am J Phys Anthropol. 2011;146 Suppl 53:99–133. Epub 2011/12/07. doi: 10.1002/ajpa.21613 . 22101689 10.1002/ajpa.21613

[13] Kolman CJ, Centurion-Lara A, Lukehart SA, Owsley DW, Tuross N. Identification of *Treponema pallidum* subspecies *pallidum* in a 200-year-old skeletal specimen. Journal of Infectious Diseases. 1999;180(6):2060–3. doi: 10.1086/315151 10558971 10.1086/315151

[14] Montiel R, Solórzano E, Díaz N, Álvarez-Sandoval BA, González-Ruiz M, Cañadas MP, et al Neonate human remains: a window of opportunity to the molecular study of ancient syphilis. PloS one. 2012;7(5):e36371 doi: 10.1371/journal.pone.0036371 22567153 10.1371/journal.pone.0036371 PMC3342265

[15] Castro R, Prieto E, Aguas MJ, Manata MJ, Botas J, Santo I, et al Detection of Treponema pallidum sp pallidum DNA in latent syphilis. International journal of STD & AIDS. 2007;18(12):842–5. Epub 2007/12/13. doi: 10.1258/095646207782716901 . 18073019 10.1258/095646207782716901

[16] von Hunnius TE, Yang D, Eng B, Waye JS, Saunders SR. Digging deeper into the limits of ancient DNA research on syphilis. Journal of Archaeological Science. 2007;34(12):2091–100.

[17] Gogarten JF, Dux A, Schuenemann VJ, Nowak K, Boesch C, Wittig RM, et al Tools for opening new chapters in the book of Treponema pallidum evolutionary history. Clinical microbiology and infection: the official publication of the European Society of Clinical Microbiology and Infectious Diseases. 2016;22(11):916–21. Epub 2016/08/09. doi: 10.1016/j.cmi.2016.07.027 . 27498082 10.1016/j.cmi.2016.07.027

[18] Márquez Morfín L, Meza Manzanilla M. Sífilis en la Ciudad de México: análisis osteopatológico. Cuicuilco. 2015;22:89–126.

[19] Escobedo Ramírez DAR, M.; Berdeja Martínez, J. A.; Gómez Martínez, A. E. Reporte General del Proyecto Arqueologico Santa Isabel. Estacionamiento Bellas Artes. Subbdireccion de Salvamento Arqueologico, INAH, Mexico City. 1995.

[20] Scheuer L, Black S. Developmental juvenile osteology Elsevier Academic Press, Oxford 2000.

[21] Ubelaker D. Human Skeletal Remains Excavation, Analysis, Interpretation. Smithoinam Institution. D Ubelaker 1978.

[22] Massler M, Schour I. Atlas of the mouth in health and disease: American Dental Association; 1958.

[23] Liversidge HM. Accuracy of age estimation from developing teeth of a population of known age (0–5.4 years). International Journal of Osteoarchaeology. 1994;4(1):37–45.

[24] Jeanty P, Romero R. Normal values for the leg. In: R Romero, G Pilu, P Jeanty, A Ghidini, J C Hobbins (eds) Prenatal Diagnosis of Congenital Anomalies Appleton and Lange: Norwalk. 1983:324.

[25] Buikstra JE, Ubelaker DH. Standards for data collection from human skeletal remains: Proceedings of a seminar at the Field Museum of Natural History (Arkansas Archaeology Research Series 44). Fayetteville Arkansas Archaeological Survey. 1994.

[26] Ortner DJ. Identification of pathological conditions in human skeletal remains Academic Press, 6 edn, London 2003.

[27] Powell ML, Cook DC. The myth of syphilis: the natural history of treponematosis in North America: University Press of Florida; 2005.

[28] Rothschild BM, Rothschild C. Treponemal disease revisited: Skeletal discriminators for Yaws, Bejel, and venereal syphilis. Clinical Infectious Diseases. 1995;20(5):1402–8. 7620034 10.1093/clinids/20.5.1402

[29] Cremin B, Fisher R. The lesions of congenital syphilis. The British journal of radiology. 1970;43(509):333–41. doi: 10.1259/0007-1285-43-509-333 5428683 10.1259/0007-1285-43-509-333

[30] Resnick D, Niwayama G. Osteomyelitis, septic arthritis, and soft tissue infection: organisms In Resnick D (ed) Diagnosis of bone and joint disorders 3rd Philadelphia: Saunders 1995:2448–558.

[31] Aufderheide AC, Rodríguez-Martín C, Langsjoen O. The Cambridge encyclopedia of human paleopathology: Cambridge University Press Cambridge; 1998.

[32] Chungara J, Banda B, Moreno O. Sífilis congénita: Presentación de un caso radiológico. Cuadernos. 2006;51:66–9.

[33] Marks M, Mitja O, Vestergaard LS, Pillay A, Knauf S, Chen CY, et al Challenges and key research questions for yaws eradication. The Lancet Infectious diseases. 2015;15(10):1220–5. Epub 2015/09/13. doi: 10.1016/S1473-3099(15)00136-X ; PubMed Central PMCID: PMCPmc4668588. 26362174 10.1016/S1473-3099(15)00136-X PMC4668588

[34] Márquez Morfín L. La sífilis y su carácter endémico en la Ciudad de México. Historia Mexicana. 2015;64:1099–162.

[35] Márquez Morfín L, Sosa Márquez L. Mortalidad de niños y sífilis congénita en la Ciudad de México en 1915. Estudios Demográficos y Urbanos. 2016;31:177–206.

[36] Nissanka-Jayasuriya EH, Odell EW, Phillips C. Dental stigmata of congenital syphilis: a historic review with present day relevance. Head and neck pathology. 2016;10(3):327–31. doi: 10.1007/s12105-016-0703-z 26897633 10.1007/s12105-016-0703-z PMC4972761

[37] Hacket C. Diagnostic Criteria of Syphilis, Yaws and Treponarid (Treponematoses) and of Some Other Diseases in Dry Bones: For Use in Osteo-Archaeology: Springer Science & Business Media; 2013.

[38] Dabney J, Knapp M, Glocke I, Gansauge MT, Weihmann A, Nickel B, et al Complete mitochondrial genome sequence of a Middle Pleistocene cave bear reconstructed from ultrashort DNA fragments. Proceedings of the National Academy of Sciences of the United States of America. 2013;110(39):15758–63. Epub 2013/09/11. doi: 10.1073/pnas.1314445110 ; PubMed Central PMCID: PMCPmc3785785. 24019490 10.1073/pnas.1314445110 PMC3785785

[39] Meyer M, Kircher M. Illumina sequencing library preparation for highly multiplexed target capture and sequencing. Cold Spring Harbor protocols. 2010;2010(6):pdb.prot5448. Epub 2010/06/03. doi: 10.1101/pdb.prot5448 . 20516186 10.1101/pdb.prot5448

[40] Kircher M, Sawyer S, Meyer M. Double indexing overcomes inaccuracies in multiplex sequencing on the Illumina platform. Nucleic acids research. 2012;40(1):e3 Epub 2011/10/25. doi: 10.1093/nar/gkr771 ; PubMed Central PMCID: PMCPmc3245947. 22021376 10.1093/nar/gkr771 PMC3245947

[41] Arora N, Schuenemann VJ, Jager G, Peltzer A, Seitz A, Herbig A, et al Origin of modern syphilis and emergence of a pandemic Treponema pallidum cluster. Nature microbiology. 2016;2:16245 Epub 2016/12/06. doi: 10.1038/nmicrobiol.2016.245 . 27918528 10.1038/nmicrobiol.2016.245

[42] Briggs AW, Stenzel U, Johnson PL, Green RE, Kelso J, Prufer K, et al Patterns of damage in genomic DNA sequences from a Neandertal. Proceedings of the National Academy of Sciences of the United States of America. 2007;104(37):14616–21. Epub 2007/08/24. doi: 10.1073/pnas.0704665104 ; PubMed Central PMCID: PMCPmc1976210. 17715061 10.1073/pnas.0704665104 PMC1976210

[43] Sawyer S, Krause J, Guschanski K, Savolainen V, Paabo S. Temporal patterns of nucleotide misincorporations and DNA fragmentation in ancient DNA. PLoS One. 2012;7(3):e34131 Epub 2012/04/06. doi: 10.1371/journal.pone.0034131 ; PubMed Central PMCID: PMCPmc3316601. 22479540 10.1371/journal.pone.0034131 PMC3316601

[44] Maricic T, Whitten M, Paabo S. Multiplexed DNA sequence capture of mitochondrial genomes using PCR products. PLoS One. 2010;5(11):e14004 Epub 2010/11/26. doi: 10.1371/journal.pone.0014004 ; PubMed Central PMCID: PMCPmc2982832. 21103372 10.1371/journal.pone.0014004 PMC2982832

[45] Renaud G, Slon V, Duggan AT, Kelso J. Schmutzi: estimation of contamination and endogenous mitochondrial consensus calling for ancient DNA. Genome biology. 2015;16:224 Epub 2015/10/16. doi: 10.1186/s13059-015-0776-0 ; PubMed Central PMCID: PMCPmc4601135. 26458810 10.1186/s13059-015-0776-0 PMC4601135

[46] Petrosova H, Zobanikova M, Cejkova D, Mikalova L, Pospisilova P, Strouhal M, et al Whole genome sequence of Treponema pallidum ssp. pallidum, strain Mexico A, suggests recombination between yaws and syphilis strains. PLoS Negl Trop Dis. 2012;6(9):e1832 Epub 2012/10/03. doi: 10.1371/journal.pntd.0001832 ; PubMed Central PMCID: PMCPmc3447947. 23029591 10.1371/journal.pntd.0001832 PMC3447947

[47] Staudova B, Strouhal M, Zobanikova M, Cejkova D, Fulton LL, Chen L, et al Whole genome sequence of the Treponema pallidum subsp. endemicum strain Bosnia A: the genome is related to yaws treponemes but contains few loci similar to syphilis treponemes. PLoS Negl Trop Dis. 2014;8(11):e3261 Epub 2014/11/07. doi: 10.1371/journal.pntd.0003261 ; PubMed Central PMCID: PMCPmc4222731. 25375929 10.1371/journal.pntd.0003261 PMC4222731

[48] Mikalová L, Strouhal M, Oppelt J, Grange PA, Janier M, Benhaddou N, et al Human Treponema pallidum 11q/j isolate belongs to subsp. endemicum but contains two loci with a sequence in TP0548 and TP0488 similar to subsp. pertenue and subsp. pallidum, respectively. PLoS neglected tropical diseases. 2017;11(3):e0005434 doi: 10.1371/journal.pntd.0005434 28263990 10.1371/journal.pntd.0005434 PMC5354452

[49] Cejkova D, Zobanikova M, Chen L, Pospisilova P, Strouhal M, Qin X, et al Whole genome sequences of three Treponema pallidum ssp. pertenue strains: yaws and syphilis treponemes differ in less than 0.2% of the genome sequence. PLoS Negl Trop Dis. 2012;6(1):e1471 Epub 2012/02/01. doi: 10.1371/journal.pntd.0001471 ; PubMed Central PMCID: PMCPmc3265458. 22292095 10.1371/journal.pntd.0001471 PMC3265458

[50] Bouckaert R, Heled J, Kuhnert D, Vaughan T, Wu CH, Xie D, et al BEAST 2: a software platform for Bayesian evolutionary analysis. PLoS computational biology. 2014;10(4):e1003537 Epub 2014/04/12. doi: 10.1371/journal.pcbi.1003537 ; PubMed Central PMCID: PMCPmc3985171. 24722319 10.1371/journal.pcbi.1003537 PMC3985171

[51] Bouckaert RR. DensiTree: making sense of sets of phylogenetic trees. Bioinformatics. 2010;26(10):1372–3. doi: 10.1093/bioinformatics/btq110 . 20228129 10.1093/bioinformatics/btq110

[52] Didelot X, Wilson DJ. ClonalFrameML: efficient inference of recombination in whole bacterial genomes. PLoS computational biology. 2015;11(2):e1004041 doi: 10.1371/journal.pcbi.1004041 ; PubMed Central PMCID: PMCPMC4326465. 25675341 10.1371/journal.pcbi.1004041 PMC4326465

[53] Schmidt HA, Strimmer K, Vingron M, von Haeseler A. TREE-PUZZLE: maximum likelihood phylogenetic analysis using quartets and parallel computing. Bioinformatics. 2002;18(3):502–4. Epub 2002/04/06. . 11934758 10.1093/bioinformatics/18.3.502

[54] Fraser CM, Norris SJ, Weinstock GM, White O, Sutton GG, Dodson R, et al Complete genome sequence of Treponema pallidum, the syphilis spirochete. Science. 1998;281(5375):375–88. 9665876 10.1126/science.281.5375.375

[55] Weinstock GM, Hardham JM, McLeod MP, Sodergren EJ, Norris SJ. The genome of Treponema pallidum: new light on the agent of syphilis. FEMS Microbiology Reviews. 1998;22(4):323–32. 9862125 10.1111/j.1574-6976.1998.tb00373.x

[56] Radolf JD, Deka RK, Anand A, Šmajs D, Norgard MV, Yang XF. Treponema pallidum, the syphilis spirochete: making a living as a stealth pathogen. Nature Reviews Microbiology. 2016;14(12):744 doi: 10.1038/nrmicro.2016.141 27721440 10.1038/nrmicro.2016.141 PMC5106329

[57] Radolf JD, Kumar S. The Treponema pallidum outer membrane. 2017. 10.1007/82_2017_44 PMC5924592 28849315

[58] Šmajs D, Norris SJ, Weinstock GM. Genetic diversity in *Treponema pallidum*: implications for pathogenesis, evolution and molecular diagnostics of syphilis and yaws. Infection, Genetics and Evolution. 2012;12(2):191–202. doi: 10.1016/j.meegid.2011.12.001 22198325 10.1016/j.meegid.2011.12.001 PMC3786143

[59] LaFond RE, Lukehart SA. Biological basis for syphilis. Clinical microbiology reviews. 2006;19(1):29–49. doi: 10.1128/CMR.19.1.29-49.2006 16418521 10.1128/CMR.19.1.29-49.2006 PMC1360276

[60] Kubanov A, Runina A, Deryabin D. Novel Treponema pallidum Recombinant Antigens for Syphilis Diagnostics: Current Status and Future Prospects. BioMed research international. 2017;2017. 10.1155/2017/1436080 PMC5421087 28523273

[61] Hearn EM, Patel DR, Van den Berg B. Outer-membrane transport of aromatic hydrocarbons as a first step in biodegradation. Proceedings of the National Academy of Sciences. 2008;105(25):8601–6. 10.1073/pnas.0801264105 PMC2438428 18559855

[62] Cox DL, Radolf JD. Insertion of fluorescent fatty acid probes into the outer membranes of the pathogenic spirochaetes Treponema pallidum and Borrelia burgdorferi. Microbiology. 2001;147(5):1161–9. 11320119 10.1099/00221287-147-5-1161

[63] Mikalová L, Strouhal M, Čejková D, Zobaníková M, Pospíšilová P, Norris SJ, et al Genome analysis of Treponema pallidum subsp. pallidum and subsp. pertenue strains: most of the genetic differences are localized in six regions. PLoS One. 2010;5(12):e15713 doi: 10.1371/journal.pone.0015713 21209953 10.1371/journal.pone.0015713 PMC3012094

[64] Roman GC, Roman LN. Occurrence of congenital, cardiovascular, visceral, neurologic, and neuro-ophthalmologic complications in late yaws: a theme for future research. Reviews of infectious diseases. 1986;8(5):760–70. Epub 1986/09/01. . 3538316

[65] Engelhardt HK. A study of yaws (does congenital yaws occur?). Journal of Tropical Medicine and Hygiene. 1959;62(10):238–40. 13820342

[66] Giacani L, Lukehart SA. The endemic treponematoses. Clinical microbiology reviews. 2014;27(1):89–115. doi: 10.1128/CMR.00070-13 24396138 10.1128/CMR.00070-13 PMC3910905

[67] Wicher K, Wicher V, Abbruscato F, Baughn RE. Treponema pallidum subsp. pertenue Displays Pathogenic Properties Different from Those of T. pallidumsubsp. pallidum. Infection and immunity. 2000;68(6):3219–25. 10816466 10.1128/iai.68.6.3219-3225.2000 PMC97566

[68] Mitja O, Asiedu K, Mabey D. Yaws. Lancet (London, England). 2013;381(9868):763–73. Epub 2013/02/19. doi: 10.1016/s0140-6736(12)62130-8 . 23415015 10.1016/S0140-6736(12)62130-8

[69] Turner TB, Hollander DH, Organization WH. Biology of the treponematoses World Health Organization, Geneva, Switzerland 1957. 13423342

[70] Edington GM. Cardiovascular disease as a cause of death in the Gold Coast African. Transactions of the Royal Society of Tropical Medicine and Hygiene. 1954;48(5):419–25. Epub 1954/09/01. . 13216946 10.1016/0035-9203(54)90143-1

[71] Achtman M. Evolution, population structure, and phylogeography of genetically monomorphic bacterial pathogens. Annual review of microbiology. 2008;62:53–70. Epub 2008/09/13. doi: 10.1146/annurev.micro.62.081307.162832 . 18785837 10.1146/annurev.micro.62.081307.162832

[72] Van Den Berg B, Black PN, Clemons WM, Rapoport TA. Crystal structure of the long-chain fatty acid transporter FadL. Science. 2004;304(5676):1506–9. doi: 10.1126/science.1097524 15178802 10.1126/science.1097524

[73] van den Berg B. The FadL family: unusual transporters for unusual substrates. Current opinion in structural biology. 2005;15(4):401–7. doi: 10.1016/j.sbi.2005.06.003 16005205 10.1016/j.sbi.2005.06.003

[74] Bouwman AS, Brown TA. The limits of biomolecular palaeopathology: ancient DNA cannot be used to study venereal syphilis. Journal of Archaeological Science. 2005;32(5):703–13.

[75] Fribourg-Blanc A, Mollaret H. Natural treponematosis of the African primate. Primates in medicine. 1969;3:113–21. 5006024

[76] Briggs AW, Stenzel U, Meyer M, Krause J, Kircher M, Paabo S. Removal of deaminated cytosines and detection of in vivo methylation in ancient DNA. Nucleic acids research. 2010;38(6):e87 Epub 2009/12/24. doi: 10.1093/nar/gkp1163 ; PubMed Central PMCID: PMCPmc2847228. 20028723 10.1093/nar/gkp1163 PMC2847228

[77] Gansauge MT, Meyer M. Single-stranded DNA library preparation for the sequencing of ancient or damaged DNA. Nature protocols. 2013;8(4):737–48. Epub 2013/03/16. doi: 10.1038/nprot.2013.038 . 23493070 10.1038/nprot.2013.038

[78] Hodges E, Rooks M, Xuan Z, Bhattacharjee A, Benjamin Gordon D, Brizuela L, et al Hybrid selection of discrete genomic intervals on custom-designed microarrays for massively parallel sequencing. Nature protocols. 2009;4(6):960–74. Epub 2009/05/30. doi: 10.1038/nprot.2009.68 ; PubMed Central PMCID: PMCPmc2990409. 19478811 10.1038/nprot.2009.68 PMC2990409

[79] Peltzer A, Jager G, Herbig A, Seitz A, Kniep C, Krause J, et al EAGER: efficient ancient genome reconstruction. Genome biology. 2016;17:60 Epub 2016/04/03. doi: 10.1186/s13059-016-0918-z ; PubMed Central PMCID: PMCPmc4815194. 27036623 10.1186/s13059-016-0918-z PMC4815194

[80] Li H, Durbin R. Fast and accurate long-read alignment with Burrows-Wheeler transform. Bioinformatics. 2010;26(5):589–95. Epub 2010/01/19. doi: 10.1093/bioinformatics/btp698 ; PubMed Central PMCID: PMCPmc2828108. 20080505 10.1093/bioinformatics/btp698 PMC2828108

[81] Broad Institute. Picard Tools version 1.140 2016.

[82] Jónsson H, Ginolhac A, Schubert M, Johnson PL, Orlando L. mapDamage2.0: fast approximate Bayesian estimates of ancient DNA damage parameters. Bioinformatics. 2013:btt193. 10.1093/bioinformatics/btt193 PMC3694634 23613487

[83] DePristo MA, Banks E, Poplin R, Garimella KV, Maguire JR, Hartl C, et al A framework for variation discovery and genotyping using next-generation DNA sequencing data. Nat Genet. 2011;43(5):491–8. Epub 2011/04/12. doi: 10.1038/ng.806 ; PubMed Central PMCID: PMCPmc3083463. 21478889 10.1038/ng.806 PMC3083463

[84] Cingolani P, Platts A, Wang le L, Coon M, Nguyen T, Wang L, et al A program for annotating and predicting the effects of single nucleotide polymorphisms, SnpEff: SNPs in the genome of Drosophila melanogaster strain w1118; iso-2; iso-3. Fly. 2012;6(2):80–92. Epub 2012/06/26. doi: 10.4161/fly.19695 ; PubMed Central PMCID: PMCPmc3679285. 22728672 10.4161/fly.19695 PMC3679285

[85] Weissensteiner H, Pacher D, Kloss-Brandstatter A, Forer L, Specht G, Bandelt HJ, et al HaploGrep 2: mitochondrial haplogroup classification in the era of high-throughput sequencing. Nucleic Acids Res. 2016;44(W1):W58–63. doi: 10.1093/nar/gkw233 ; PubMed Central PMCID: PMCPMC4987869. 27084951 10.1093/nar/gkw233 PMC4987869

[86] Stamatakis A. RAxML version 8: a tool for phylogenetic analysis and post-analysis of large phylogenies. Bioinformatics. 2014;30(9):1312–3. Epub 2014/01/24. doi: 10.1093/bioinformatics/btu033 ; PubMed Central PMCID: PMCPmc3998144. 24451623 10.1093/bioinformatics/btu033 PMC3998144

[87] Kumar S, Stecher G, Tamura K. MEGA7: Molecular Evolutionary Genetics Analysis Version 7.0 for Bigger Datasets. Molecular biology and evolution. 2016;33(7):1870–4. doi: 10.1093/molbev/msw054 . 27004904 10.1093/molbev/msw054 PMC8210823

[88] Rambaut AS, M.A.; Xie, D.; Drummond, A.J. Tracer v1.6, Available from http://beast.bio.ed.ac.uk/Tracer. 2014.

[89] Quinlan AR, Hall IM. BEDTools: a flexible suite of utilities for comparing genomic features. Bioinformatics. 2010;26(6):841–2. doi: 10.1093/bioinformatics/btq033 20110278 10.1093/bioinformatics/btq033 PMC2832824

[90] Krzywinski M, Schein J, Birol I, Connors J, Gascoyne R, Horsman D, et al Circos: an information aesthetic for comparative genomics. Genome research. 2009;19(9):1639–45. doi: 10.1101/gr.092759.109 19541911 10.1101/gr.092759.109 PMC2752132

