# Supplementary figures and images for: Historic Treponema pallidum genomes from Colonial Mexico retrieved from archaeological remains
Source: PLoS Negl Trop Dis. 2018 Jun 21;12(6):e0006447. doi: 10.1371/journal.pntd.0006447 (PMC6013024; doi:10.1371/journal.pntd.0006447)

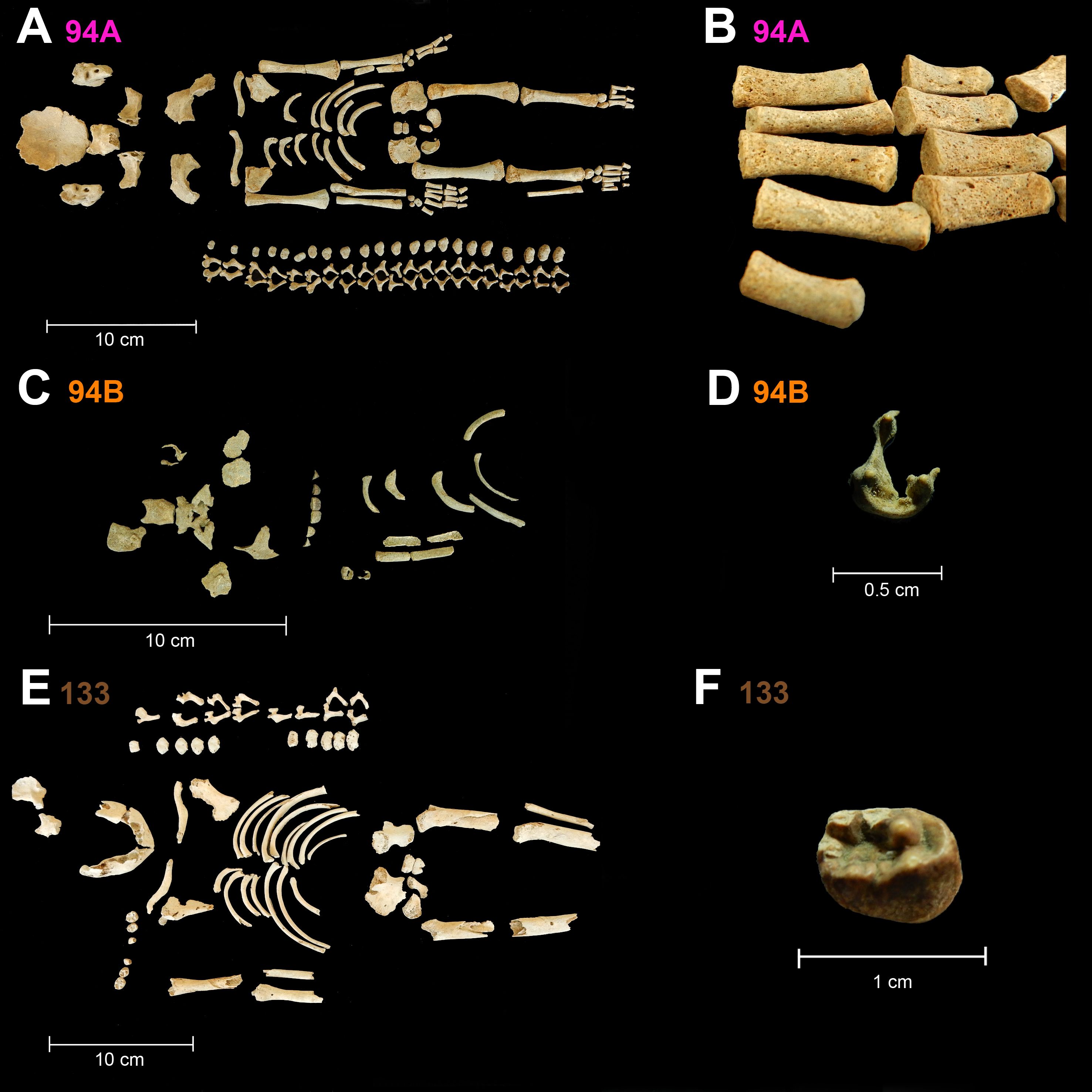

Supplement: S1 Fig — (A) Complete skeletal elements for individual 94A. (B) Metacarpals and phalanges of individual 94A displaying periostitis. (C) Complete skeletal elements for individual 94B. (D) Fragment of the still developing molar crown (at time of death) of individual 94B. (E) Complete skeletal elements for individual 133. (F) First mandibular molar of individual 133 displaying normal crown development. Source of the pictures: skeletal collection from Santa Isabel Convent, Mexico City, in custody of the Laboratory of Osteology, Post Graduate Studies Division, National School of Anthropology and History (ENAH), Mexico. (JPEG) [file pntd.0006447.s001.jpeg]

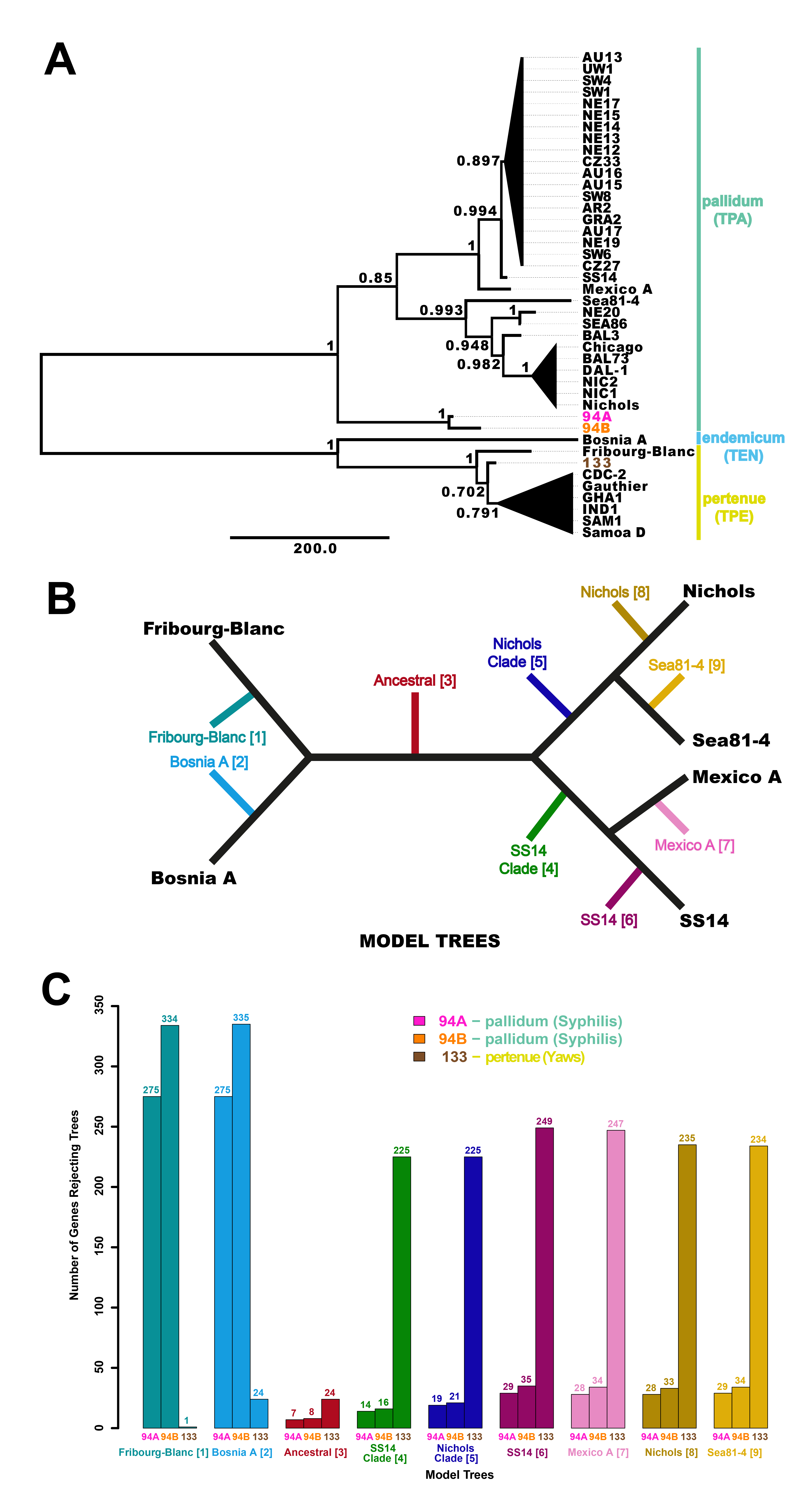

Supplement: S2 Fig — (A) Maximum Parsimony tree with bootstrap support for 39 modern genomes and the three ancient samples. The scale bar represents the number of mutations over the whole genome calculated by average pathway method. Colored bars highlight the three subspecies Treponema pallidum subspecies pallidum (TPA), pertenue (TPE) and endemicum (TEN). Strains of subspecies pallidum cause syphilis, subspecies pertenue causes yaws and subspecies endemicum causes bejel. (B) The nine model trees used in the TREE-PUZZLE analysis. The colored edges represent the branching of the ancient sample with specific representative strains that are denoted by their names on the tree topology backbone. The red edge represents an ancestral branching of the historic sample while the green edge denotes the branching of the sample with the SS14 clade and the blue edge denotes the branching of the sample with the Nichols clade. The number in parenthesis represents tree topology model. (C) Bar plot representing the number of genes of the historic strains 94A, 94B and 133 that reject specific tree topologies. Each plot represents a specific tree model for 94A (magenta), 94B (orange) and 133 (brown) and the bar colors correspond to the color scheme as in S2B Fig. (PNG) [file pntd.0006447.s002.png]

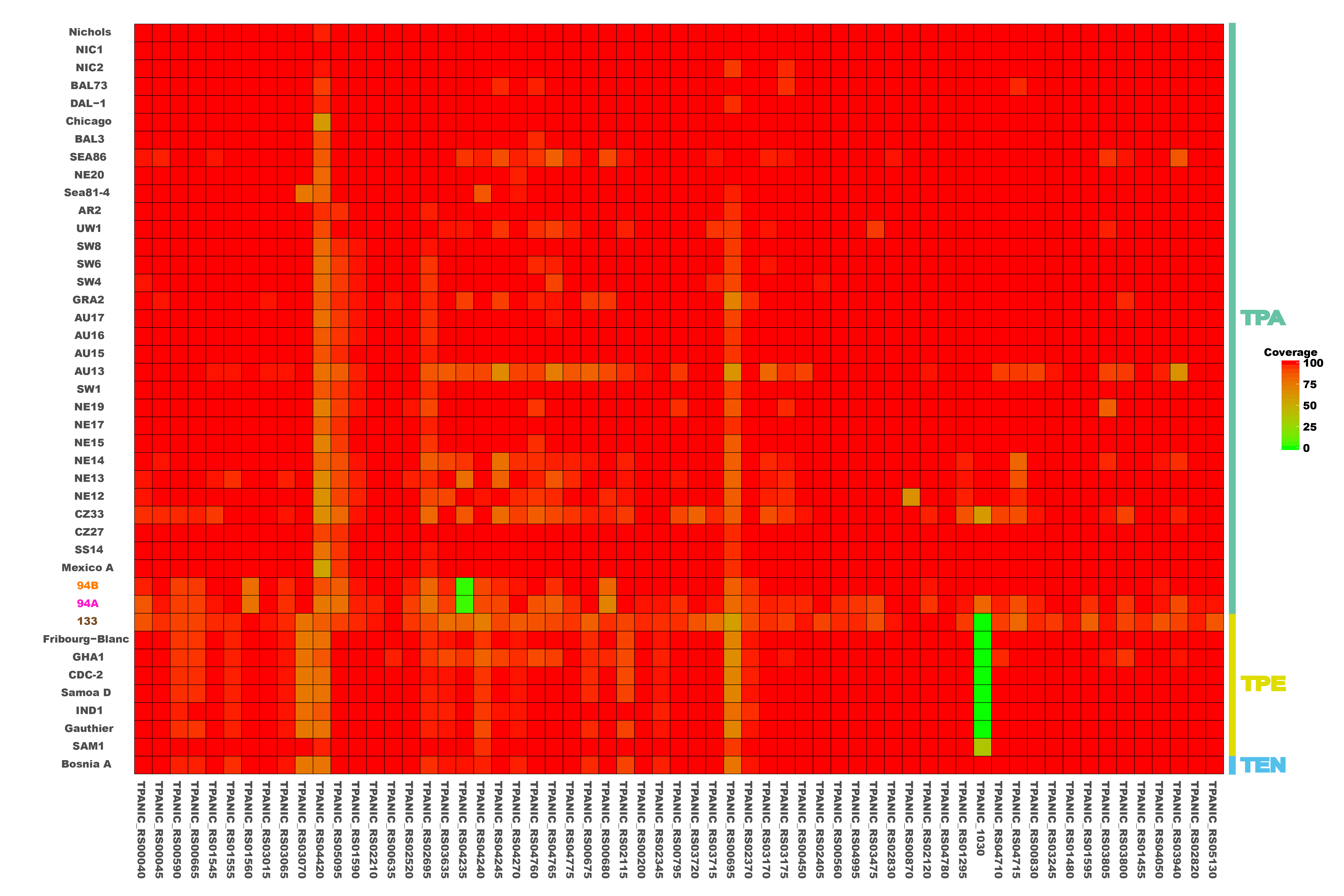

Supplement: S3 Fig — The heatmap summarizes the presence and absence of various virulence factors for the ancient and modern genomes in terms of gene coverage. The color key represents the percentage of coverage. Red represents gene presence while green represents gene absence. The ancient strains 94A (magenta), 94B (orange) and 133 (brown) are highlighted. Colored bars represent the three subspecies subsp. pallidum (TPA), subsp. pertenue (TPE) and subsp. endemicum (TEN). (PNG) [file pntd.0006447.s003.png]
